# Improved Hydrolytic and Mechanical Stability of Sulfonated Aromatic Proton Exchange Membranes Reinforced by Electrospun PPSU Fibers

**DOI:** 10.3390/membranes12111159

**Published:** 2022-11-17

**Authors:** Luca Pasquini, Maxime Sauvan, Riccardo Narducci, Emanuela Sgreccia, Philippe Knauth, Maria Luisa Di Vona

**Affiliations:** 1 CNRS, MADIREL UMR 7246 (ELMA Team) and International Laboratory “Ionomer Materials for Energy” (LIME), Aix-Marseille University, 13013 Marseille, France; 2 International Laboratory “Ionomer Materials for Energy” (LIME), Department of Industrial Engineering, University of Rome Tor Vergata, 00133 Rome, Italy

**Keywords:** ionomer membrane, electrospinning, Young’s modulus, water uptake, ionic conductivity, fuel cells, enzymatic fuel cells, PEM fuel cells

## Abstract

The hydrolytic stability of ionomer membranes is a matter of concern for the long-term durability of energy storage and conversion devices. Various reinforcement strategies exist for the improvement of the performances of the overall membrane. We propose in this article the stabilization of membranes based on aromatic ion conducting polymers (SPEEK and SPPSU) by the introduction of an electrospun mat of inexpensive PPSU polymer. Characterization data from hydrolytic stability (mass uptake and dimension change) and from mechanical and conductivity measurements show an improved stability of membranes in phosphate buffer, used for enzymatic fuel cells, and in distilled water. The synergistic effect of the reinforcement, together with the casting solvent and the thermal treatment or blending polymers, is promising for the realization of high stability ionomer membranes.

## 1. Introduction

Important properties of ionomeric membranes, including hydrolytic stability, ionic conductivity, mechanical properties, can be improved by different reinforcement techniques [1,2,3], such as the introduction of fibers of a polymer [4], the dispersion of inorganic particles [5,6], the cross-linking of polymer chains by heat treatment [7] or the blend of two polymers with complementary properties [8,9]. The membrane reinforcement is particularly attractive for the thickness reduction of commercial membranes, which is advantageous from various points of view: reduction of the quantity of ionomer and therefore of the membrane price and reduction of the total device resistance and size, which can be interesting for microdevices. At the same time, the water management and the mechanical strength of the membrane is assured and even improved [10]. Typical reinforcing materials are polymer fibers, such as polytetrafluoroethylene (PTFE), polyvinylidenefluoride (PVDF) or polyethylene (PE) [11]. As regards the addition of fibers of these non-conducting polymers, a compromise must be found between the improvement of the mechanical properties, the hydrolytic stability and the maintaining of a good ionic conductivity; it is therefore necessary to play with the optimal proportions of components to find the best possible membrane composition [12]. The most common reinforcements consist in non-woven fabric [13]. The best example is represented by the reinforced membrane developed by W. L. Gore (Gore-Select) consisting of 20 wt% of a porous PTFE matrix filled with a perfluorosulfonic acid (PFSA) solution [10]. A different strategy is to add fibers of an ion-conducting polymer; in this way the ionic conductivity is preserved or improved, but the control of the hydrolytic stability becomes more complex [12,14,15]. In this case, the diameter of the fibers is important for the conductivity: when it decreases, the conductivity increases because the nanofibers anisotropically orient the ion aggregates along their direction axis, while they are isotropically oriented in the ionomer matrix [16].

The polymer fibers can be produced by electrospinning that is a fascinating manufacturing method often used to produce fibers with a diameter of a few hundred nanometers [17,18] by applying a voltage (5–30 kV or more) between the needle of a syringe containing a polymer solution and the collector on which the polymer fibers are deposited [19]. Several parameters influence the final result: the concentration and viscosity of the polymer solution, the pumping speed of the syringe, the solvent used and in particular its volatility, the voltage applied, the syringe-collector distance, the relative humidity and the temperature of the enclosure of the electrospinning device. The polymer solution must have adequate viscosity to be able to form fibers [20]. By varying the different parameters it is possible to control the diameter of the fibers [21].

The produced fibers can be subsequently added to the polymer matrix as reinforcement by casting, allowing for the ionomeric membrane to be stabilized [22]. The reinforcement creates a net retaining of macromolecules between its meshes when the membrane is hydrated. This hydrolytic stabilization is often accompanied by an improvement in the mechanical properties [14,15,23,24,25].

Another stabilization method is the cross-linking of the polymer. For sulfonated aromatic polymers the reticulation is achieved by forming covalent SO_2_ bridges between macromolecular chains under the action of a heat treatment. The use of dimethyl sulfoxide (DMSO) as a casting solvent during the preparation of membranes facilitates the cross-linking of the polymer chains [7,26,27,28]. This strategy can also be exploited for sulfonated poly (phenyl sulfone) (SPPSU) membranes reinforced with poly (phenyl sulfone) (PPSU) fibers: in addition to create SO_2_ bonds between SPPSU chains, this method also allows for the formation of bonds between SPPSU and PPSU.

Finally, another reinforcement technique is the blend method. In our case it consists in mixing a polymer with a high ionic conductivity but a low hydrolytic stability (SPPSU) with a polymer having a lower ionic conductivity but a better hydrolytic stability (sulfonated poly (ether ether ketone)—SPEEK) to optimize the overall properties of the blend membrane [8,29].

Previously, we compared reference membranes, Nafion and SPEEK made with the DMSO casting procedure, with blend membranes or membranes cast from other solvents showing the possibility to tune the properties and stabilize the hydrolytic behavior of the ionomer [30].

In this article, electrospun fibers of PPSU are used as reinforcement for sulfonated aromatic polymers. The possible decrease in the ionic conductivity of the composite, due to the presence of the non-conductive PPSU phase, must be mitigated by a careful choice of the optimal percentage of reinforcement in order to combine high hydrolytic and mechanical stability with good ionic conductivity. These membranes might be used in PEM fuel cells of enzymatic fuel cells (EFCs), which are promising sustainable power generation systems for various applications. Their particularity is the replacement of non-selective metal catalysts, currently used in low-temperature FCs, by redox-active enzymes.

## 2. Materials and Methods

The solvents used were dimethyl sulfoxide (DMSO), N-methyl-pyrrolidone (NMP), acetone, dimethyl formamide (DMF) and ethanol, all being anhydrous and purchased from Merck-Sigma-Aldrich. Poly (ether ether ketone) (PEEK) was received from Victrex (MW = 38,300 g/mol) and poly (phenyl sulfone) (PPSU) was received from Solvay (MW = 46,173 g/mol). The sulfonated poly (ether ketone) (SPEEK) and the sulfonated poly (phenyl sulfone) (SPPSU) were prepared according to the procedures reported in references [31,32,33]. Their respective degrees of functionalization (degree of sulfonation DS) and ion exchange capacity (IEC), determined by NMR or titration [34], were 92% and 2.50 meq/g (SPEEK) and 152% and 2.92 meq/g (SPPSU). The choice of SPEEK and SPPSU polymers allows for exploiting the properties of each polymer: the higher hydrolytic stability but lower ionic conductivity (lower DS and IEC) of the former with the higher conductivity but lower hydrolytic stability of the latter (higher DS and IEC).

### 2.1. Electrospinning

The electrospinning apparatus consisted in a Starter Kit-Random equipment (Linari Engineering SRL, Pise, Italy). A 10 mL syringe with a 0.8 mm diameter needle was mounted on the syringe pump.

The PPSU polymer reinforcements were prepared by readaptation of electrospinning parameters of references [14,35]. Many tests were carried out to optimize the reinforcement by playing on the different parameters. The final setting flow rate was 0.2 mL/h and the applied voltage 12 kV with a tip-collector distance of 17 cm. The temperature of the enclosure was of 25 ± 2 °C and the relative humidity 40 ± 5%. The mat size was 18 × 18 cm^2^ with a thickness of 27 ± 5 μm

### 2.2. Membrane Casting

All membranes were cast from an 8 wt% polymer solution in a Petri dish and heated in an oven at 80 °C for 18 h in order to evaporate the solvents (ethanol, water, DMSO or a mixture). The choice of the polymer/solvent coupling and some data of non-reinforced membranes were taken from reference [30]. DMSO was used as a solvent to allow the cross-linking of the polymer chains by a heat treatment following casting, as previously described [26]. The electrospun PPSU reinforcement mat was impregnated with the casting solution and the standard casting procedure was applied. The percentage of reinforcement in the membrane was 10 ± 1 wt%.

### 2.3. Scanning Electron Microscopy

The scanning electron microscope utilized to observe the morphology of the fibers produced by electrospinning and the section of the reinforced membranes before and after the hydrolytic swelling was a Zeiss GeminiSEM 500. For the measurements, a low voltage (2–5 kV) was used because the samples are electronically insulating. To analyze the section of the reinforced membranes, the sample was cut in liquid nitrogen to have a sharp surface. The micrographs of the electrospun fiber mats were analyzed with the ImageJ software to evaluate the distribution of the fiber diameters.

### 2.4. Mechanical Test

The mechanical properties of the membranes were studied with a Testometric M250 -2.5 CT tensile machine (Testometric, Rochdale, UK) by plotting the tensile curve stress versus strain; Young’s modulus was determined from the slope of the linear part of the curve. The samples were cut (being careful to have sharp cuts) with a rectangular shape of 5 mm in width and approximately 50 mm in length (for an active length of 25 mm). The thickness was measured at 5 points of the sample with a Mitutoyo 293–230 micrometer. Then, the sample was placed in the traction machine by mechanical clamps. The experiment was conducted with a pulling speed of 5 mm/min. 2 samples for each membrane were tested to perform an average. The temperature during the test was 25 ± 2 °C and the humidity 40 ± 10%.

### 2.5. Hydrolytic Stability

The hydrolytic stability was investigated in a 0.05 M potassium phosphate buffer (H_2_PO_4_^−^/HPO_4_^2−^) at pH = 6.5 ± 0.2 or in distilled water. The pH was determined with a calibrated pH-meter OrigaMeter OpH218 (OrigaLys, Rillieux-la-Pape, France). This buffer was chosen because, in the perspective of the use of these membranes in biological fuel cells, it stabilizes most of the enzymes in enzymatic fuel cells, in particular bilirubin oxidase [36,37].

Two essential hydrolytic stability parameters were investigated: the mass uptake *MU* and the dimensional change Δ*dim* (*dim* = *V* for volume, *A* for area, *th* for thickness). These parameters are calculated from the mass and dimensions of the dry and wet samples, according to Equation (1):

(1) $$MU\left(\%\right)=\frac{M_{wet}-M_{dry}}{M_{dry}}\times 100\;\Delta dim\left(\%\right)=\frac{dim_{wet}-dim_{dry}}{dim_{dry}}\times 100$$

The samples were cut in a square shape of 1.5 cm side. The data of the wet samples were determined after immersion in the buffer solution during 24 h at 25 °C in a thermoregulated oven without any additional washing in water. Before the measurement, the membrane was wiped carefully with absorbing paper to remove the excess of buffer solution on the surface. The data of the dry samples were measured in a closed vessel after drying over P_2_O_5_ for 24 h. The uncertainty on the mass was 0.1 mg and for the thickness 0.1 mm. The dry density of the ionomer was determined using the mass and dimensions of the membranes after the drying process.

### 2.6. Impedance Spectroscopy

The through-plane ionic conductivity of the membranes was measured by electrochemical impedance spectroscopy between 1 Hz and 6 MHz using a Biologic VSP300 (Biologic, Seyssinet-Pariset, France). The amplitude of the sinusoidal voltage was 20 mV. After immersion in the phosphate buffer solution, the sample was carefully wiped and the measurement was carried out in a Swagelock cell with two stainless steel electrodes (area *A* = 0.264 cm^2^) at 25 °C. Some ions sorbed from the buffer solution can contribute to the overall ionic conductivity.

The resistance of the sample *R* was obtained from the intercept with the real axis in a Nyquist impedance plot as already reported in reference [30]. The conductivity of the membrane was calculated using Equation (2):

(2) $$\sigma =\frac{th}{R\cdot A}$$

*th* is the membrane thickness, determined using the Mitutoyo micrometer.

The conductivity was measured on membranes in K^+^ form.

## 3. Results and Discussion

### 3.1. Electrospinning of PPSU and Reinforced Membrane Casting

**Figure 1:**
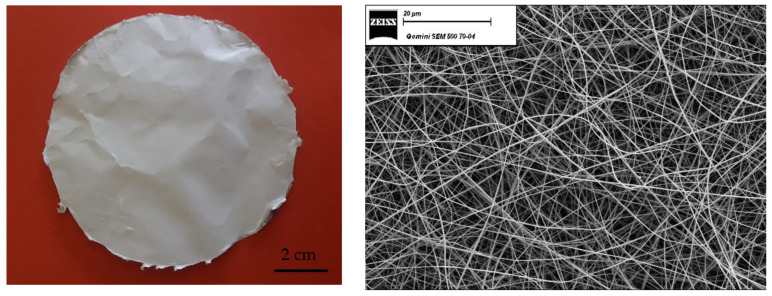
Optical and SEM micrographs (magnification 1000×) of the PPSU electrospun mat.

Figure 1 shows optical and SEM micrographs of the PPSU electrospun mat. The electrospinning solution had a composition of 23 wt% PPSU and 77 wt% solvent, which was a mixture of 75 wt% NMP and 25 wt% acetone.

**Figure 2:**
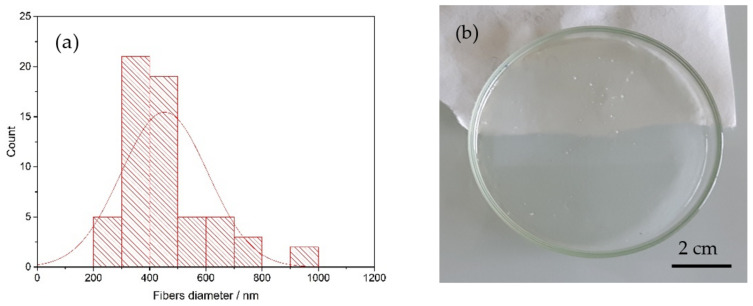
(**a**) Fibers diameter distribution of the PPSU electrospun mat and (**b**) a reinforced SPEEK membrane casted from ethanol and water (K-EW_R).

The fibers diameter distribution (reported in Figure 2a) was obtained with the program ImageJ and corresponded to an average of 450 nm. An example of reinforced membrane is reported in Figure 2b.

The different types of membranes are reported in Table 1, the final electrospun percentage inside the reinforced membranes is 10 ± 1 wt%. The membranes are named as follows: utilized polymer (K = SPEEK, U = SPPSU) with eventually the composition ratio, utilized casting solvent or solvent mixture (E = Ethanol, W = water, D = DMSO), _R indicates the presence of a PPSU reinforcement, _TT indicates a thermal treatment at 180 °C for 3 h.

**Figure 3:**
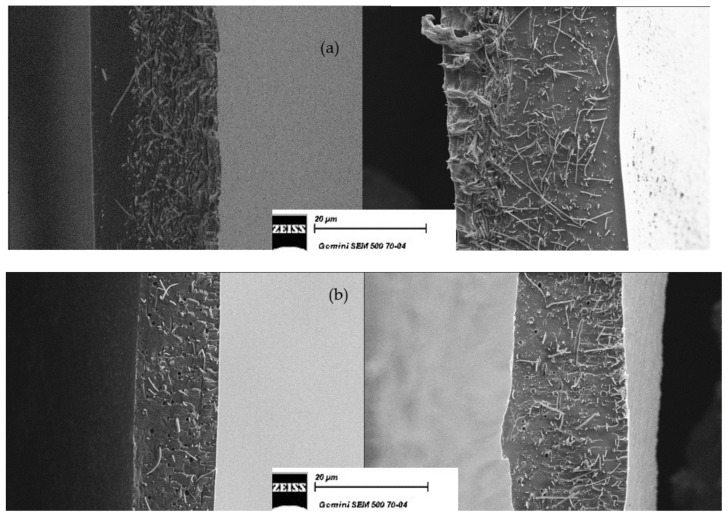
Cross-sectional SEM micrographs of reinforced membranes before (**left**) and after (**right**) the swelling in buffer. (**a**) K-EW_R and (**b**) U-E_R_TT. Magnification: 1000×.

Cross-sectional SEM images are reported in Figure 3. It is easy to observe that the fibers mat is homogeneously dispersed over the entire thickness of the membrane, which guarantees a uniform reinforcement and avoids the deformation and delamination, because the matrix polymer (SPEEK or SPPSU) is trapped in the PPSU fibers network. Figure 3 (right) reports similar observations after 24 h in the buffer solution; no modification is observed on the positioning of the fibers in the matrix. The larger swelling of a SPEEK membrane cast in the presence of water can be qualitatively observed.

### 3.2. Hydrolytic Stability of Membranes

Five main groups of membranes were analyzed differing by the manufacturing process and the combination of solvent and/or polymers. The hydrolytic stability data (mass uptake and dimensional change) as well as the Young’s modulus and the ionic conductivity of reinforced membranes are compared to the pristine non reinforced membrane.

#### 3.2.1. SPEEK Cast from Mixture 50% Ethanol and 50% Water (K-EW)

**Figure 4:**
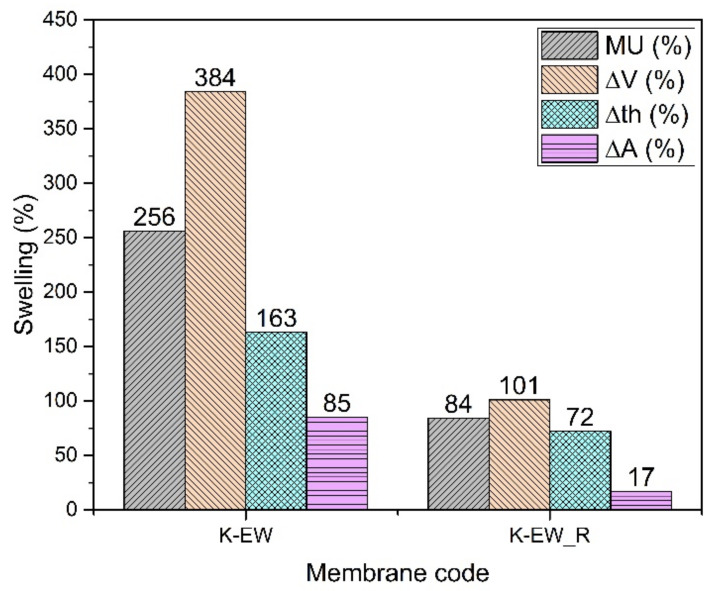
Swelling data of K-EW membranes.

The histogram (Figure 4) shows impressively the improvement of the hydrolytic stability of reinforced membranes (K-EW_R). The mass uptake and the dimensional changes (V, th, A) are lower by 67%, 74%, 56% and 80% with PPSU nanofibers, respectively. The reinforcement has a particularly significant effect on the change in the area of the membrane [11]. Since the fibers are partially aligned on the surface, the membrane dimensional stabilization is better in this dimension compared to the perpendicular one (thickness), where the fibers of the reinforcement are not bound together and are free to move. Consistently, the Young’s modulus is higher in the case of the K-EW_R membrane (Table 2). The ionic conductivity remains nearly constant or even slightly increases (26 mS/cm against 23 mS/cm) for the reinforced membranes, although the filler is not conductive. This effect may be related to a better connectivity of the ion conduction channels. The decrease in dry density can be attributed to an increase in the free volume due to the presence of the fibers.

#### 3.2.2. SPEEK Cast from Mixture 90% Water and 10% Dimethyl Sulfoxide (K-WD)

The thermal treatment (TT) at 180 °C for 3 h strongly stabilizes the membrane even without the reinforcement, probably due to some cross-linking as already shown in the references [7,25,26]: the cross-linking of chains improves the hydrolytic stability but causes a slight reduction in conductivity that is lowered both for the non-reinforced and reinforced membranes.

**Figure 5:**
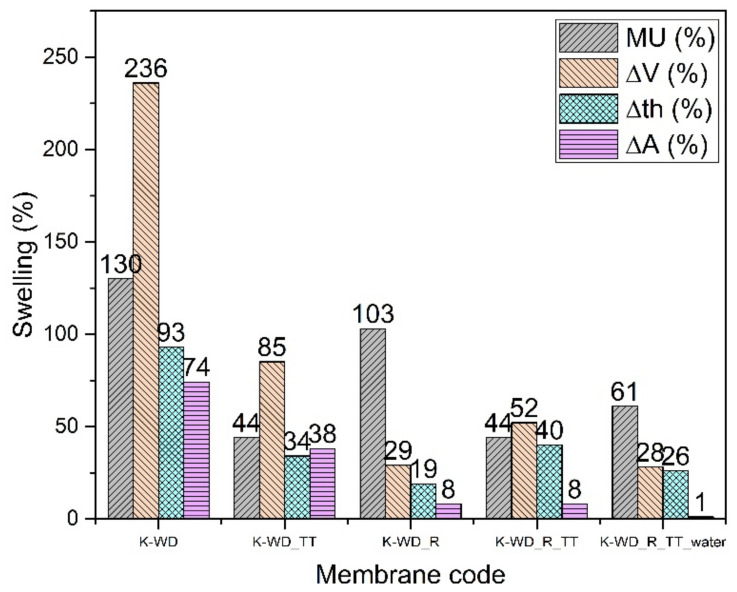
Swelling data of K-WD membranes.

The addition of PPSU fibers allows a further improvement of the hydrolytic and especially of the dimensional stability (Figure 5). Some mass uptake data (K-WD_R and K-WD_R_TT) are somehow higher than expected and probably related to the different dry density. The density reduction of reinforced membranes points again to a probable porosity increase around the fibers [38]. This hypothesis can also explain the lower Young’s modulus of the reinforced membranes (Table 3). An interesting data is the stability in pure water for the reinforced thermally treated membrane indicating great promise for its use in fuel cells.

#### 3.2.3. SPPSU Cast from Ethanol (U-E)

**Figure 6:**
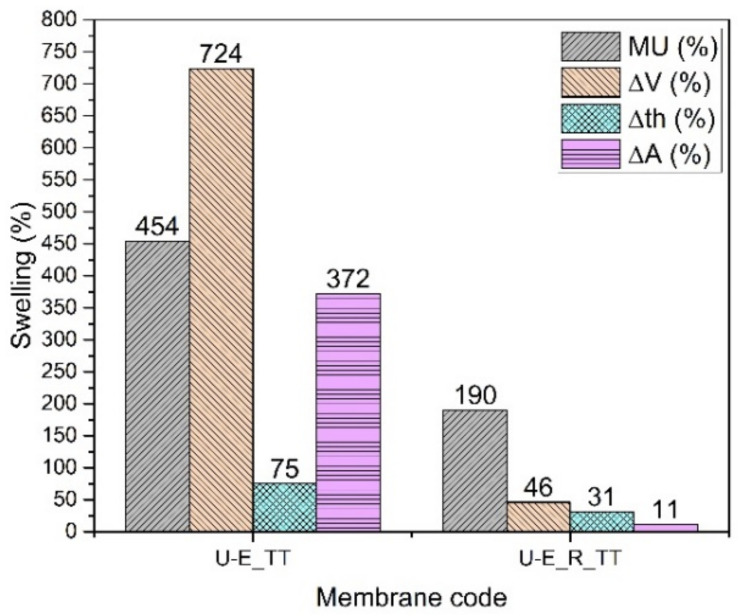
Swelling data of U-E membranes.

The SPPSU membranes made from ethanol (Figure 6) show a great hydrolytic stabilization when reinforced and annealed (U-E_R_TT). The pristine (U-E) and reinforced membranes (U-E_R), which are made with a highly functionalized polymer (DS = 152%), dissolve in the buffer solution. After an annealing treatment at 180 °C for 3 hours, the thermally treated pristine membrane (U-E_TT) is slightly stabilized, but the reinforced membrane presents a strong hydrolytic stabilization. In addition, in this case, we observe a smaller change in the area compared to the thickness, which confirms the anisotropic change following the inclusion of the electrospun reinforcement. The conductivity of the membrane (Table 4) remains quite high, due to the highly functionalized polymer and a high mass uptake because a large quantity of water increases the proton mobility [39,40]. The Young’s modulus, as expected, increases after the heat treatment and fiber reinforcement, and the dry density decreases slightly, indicating that with this casting solvent, the presence of fibers induces a limited increase in porosity.

#### 3.2.4. SPPSU Cast from Mixture 90% Water and 10% DMSO (U-WD)

**Figure 7:**
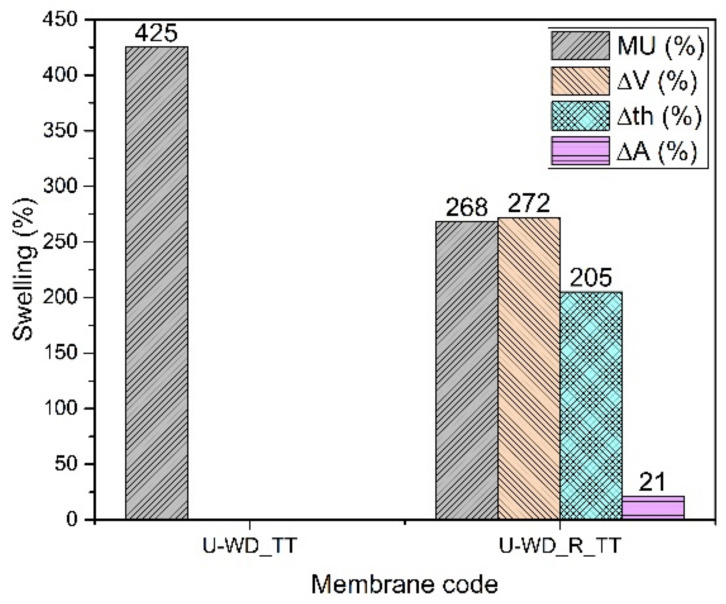
Swelling data of U-WD membranes.

Membranes composed of SPPSU from water/DMSO mixture are not stable, neither in a buffer solution nor in water and even with the reinforcement by PPSU fibers (U-WD and U-WD_R). They need a supplementary thermal treatment. However, the histogram of Figure 7 shows that the thermally treated membrane without reinforcement (U-WD_TT) is not very stable; indeed it is impossible to evaluate its dimensional or mechanical stability because it tends to break. The addition of the PPSU mat greatly improves the stability, probably because the fibers confine the polymer matrix all over the volume. In this case, the thermal treatment probably allows for a cross-linking both between SPPSU chains and between SPPSU and PPSU fibers. The mass uptake and the dimensional changes remain however relatively high following the same tendency observed before for K-WD membranes. Mechanical tests show for the reinforced membrane a Young’s modulus of 880 ± 120 MPa. Because of the cross-linking that removes sulfonic acid groups, the ionic conductivity reaches only a value of 18 mS/cm.

#### 3.2.5. Blend Membranes of 70% SPEEK and 30% SPPSU Cast from Mixture 90% Water and 10% DMSO (7K3U-WD)

**Figure 8:**
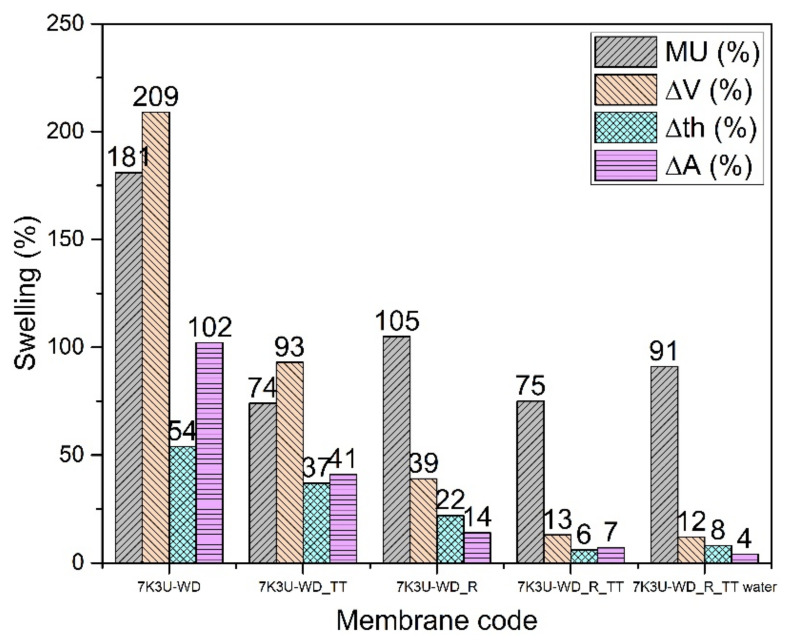
Swelling data of 7K3U-WD membranes.

The hydrolytic stability of the reinforced membranes (7K3U-WD_R and 7K3U-WD_R_TT) is much enhanced (Figure 8); a thermal treatment further improves the properties. The best compromise in this case is the 7K3U-WD_R_TT membrane, which has a good conductivity in buffer (17 mS/cm) and a low mass uptake and dimensional change. The tendency to obtain relatively high values of mass uptake for the reinforced membranes with a reduction of the dry density and of Young’s modulus can be ascribed to the same phenomenon described for the K-WD membranes (Table 5). The 7K3U-WD_R_TT membrane is also stable in water with a conductivity of 11 mS/cm, and it shows great promise for use in PEM fuel cells.

### 3.3. Discussion of Solvent Effects

As demonstrated in [30] the change of the casting solvent greatly affects the final density of the membrane and its hydrolytic stability. Solvent effects can be rationalized based on two major solvent properties: the dielectric constant and the volatility.

The dielectric constant of the casting solvent modifies the electrostatic interactions between ions and dipoles. Among the used solvents, the dielectric constant (at 25 °C) decreases in the order: water (78) > DMSO (47) > ethanol (24) [41,42,43,44]. Water leads to a clustering of ionic domains with formation of a nanophase separated morphology of membranes enhancing the water uptake and reducing the dimensional stability but also boosting the ionic conductivity.

The volatility of the solvent increases according to the boiling point at atmospheric pressure in the following order: DMSO (189 °C) < water (100 °C) < ethanol (78 °C) [45]. A residual quantity of high boiling point solvent DMSO in the membranes after casting has a plasticizing effect because the dipolar interactions between polymer chains are reduced: the Young’s modulus is lower and the water uptake higher. However, the presence of residual DMSO in the membranes allows a certain amount of cross-linking by a thermal treatment above 160 °C, as previously shown [26,27]. The formation of cross-linking SO_2_ bridges reduces the amount of sulfonic groups and therefore the ionic conductivity but leads to a significant mechanical and hydrolytic stabilization of the membranes.

The optimization of these solvent effects leads to membranes with a good compromise of the relevant properties, as shown in the case of 7K3U-WD_R_TT that presents a combination of good ionic conductivity, low swelling and high stiffness. Following the ex situ tests, these membranes are ready to be assessed in fuel cells.

## 4. Conclusions

The reinforcement by inexpensive PPSU fibers allows for a marked improvement of the properties of ionomer membranes, with a reduction in the mass uptake and dimensional change in phosphate buffer solution and in water, without a strong decrease of the ionic conductivity due to the addition of a second non-conductive polymer. The introduction of reinforcing fibers (10 wt%) leads in all cases to a decrease of the membrane density, due to the formation of free volume near the fibers. A thermal treatment of membranes gives an even better hydrolytic stability but lowers the conductivity probably because this treatment consumes sulfonic groups responsible for the ionic conductivity.

The effect of the casting solvent is also discussed based on the dielectric constant and the volatility. Casting in water leads to the formation of a nanophase separated morphology with high water uptake, low dimensional stability and high ionic conductivity; here, the reinforcement by electrospun fibers is paramount. The presence of DMSO allows the formation of cross-linking. Furthermore, due to the high boiling point of DMSO and the low casting temperature of membranes, the residual solvent contained in the system acts as a plasticizer. In ethanol/water this effect is not observed and the reinforcement and heat treatment leads to membranes that are particularly attractive for applications.

The combination of fiber reinforcement, blending of two polymers and/or thermal treatment allows for obtaining membranes that are stable in distilled water. The reinforcement of the membranes by electrospun PPSU fibers is thus an effective method to improve the membrane performances for fuel cell applications.

## Figures and Tables

**Table 1:** List of casted membranes.

Code	Polymer	Casting Solvent	Reinforcement	Thermal Treatment
K-EW	SPEEK	50% ethanol/50% water		
K-EW_R			✓	
K-WD		90% water/10% DMSO		
K-WD_TT				✓
K-WD_R			✓	
K-WD_R_TT			✓	✓
U-E	SPPSU	Ethanol		
U-E_TT				✓
U-E_R			✓	
U-E_R_TT			✓	✓
U-WD		90% water/10% DMSO		
U-WD_TT				✓
U-WD_R			✓	
U-WD_R_TT			✓	✓
7K3U-WD	70% SPEEK/30% SPPSU	90% water/10% DMSO		
7K3U-WD_TT				✓
7K3U-WD_R			✓	
7K3U-WD_R_TT			✓	✓

**Table 2:** Fully hydrated thickness, Young’s modulus, ionic conductivity and dry density of K-EW membranes.

Membrane Code	K-EW	K-EW_R
Thickness (μm)	98	78
Young’s modulus (MPa)	1090 ± 5	1130 ± 50
Ionic conductivity (mS/cm)	23	26
Dry density (g/cm^3^)	1.4	1.1

**Table 3:** Fully hydrated thickness, Young’s modulus, ionic conductivity and dry density of K-WD membranes.

Membrane Code	K-WD	K-WD_TT	K-WD_R	K-WD_R_TT
Thickness (μm)	54	40	75	73
Young’s modulus (MPa)	1080 ± 40	930 ± 120	580 ± 40	720 ± 6
Ionic conductivity (mS/cm)	16	15	13	9
Dry density (g/cm^3^)	1.5	1.4	0.6	1

**Table 4:** Fully hydrated thickness, Young’s modulus, ionic conductivity and dry density U-E membranes. * Measurement carried out in anhydrous conditions due to the solubility of the polymer.

Membrane Code	U-E	U-E_TT	U-E_R	U-E_R_TT
Thickness (μm)	41 *	63	50 *	57
Young’s modulus (MPa)	500 ± 40	--	--	860 ± 30
Ionic conductivity (mS/cm)	--	--	--	35
Dry density (g/cm^3^)	1.3	1.3	--	1.1

**Table 5:** Fully hydrated thickness, Young’s Modulus, ionic conductivity and dry density of 7K3U-WD membranes.

Membrane Code	7K3U-WD	7K3U-WD_TT	7K3U-WD_R	7K3U-WD_R_TT
Thickness (μm)	92	58	66	73
Young’s modulus (MPa)	830 ± 120	--	540 ± 10	800 ± 40
Ionic conductivity (mS/cm)	29	10	21	17
Dry density (g/cm^3^)	1.2	1.3	0.9	0.8

## Data Availability

Not applicable.
